# Supplementary material for: PanelAppRex aggregates disease gene panels and facilitates sophisticated search
Source: Bioinform Adv. 2026 May 14;6(1):vbag115. doi: 10.1093/bioadv/vbag115 (PMC13563085; doi:10.1093/bioadv/vbag115)
Supplement: Supplementary file 1 [file vbag115_supplementary_data.zip › panelapprex2025lawless_supplemental.pdf]

Supplemental: PanelAppRex aggregates disease gene panels and facilitates sophisticated search

Acronyms

API Application Programming Interface 5

GE Genomics England 5

IEI Inborn Errors of Immunity

IEM Inborn Errors of Metabolism

MOI Mode of Inheritance

PID Primary Immunodeficiency

RAG Retrieval-Augmented Generation 7

Tables

Table S1: **Summary of case study queries and PanelAppRex results.** We recommend automated use that accounts for the union of all query results. For manual interface use, judgement-based panel selection is naturally expected. We therefore recorded the user-selected best-fit panel(s), defined as the returned panel or panels judged most clinically aligned with the pre-genetic case description and most likely to be prioritised by a user. In this context, broader but less specific panels (for example, "COVID-19 research" or "Paediatric disorders") may be deprioritised relative to panels whose title and scope more closely match the presenting condition. The results are summarised in **Figure 1**. Supplemental data file: *Table S1 supplemental benchmark.tsv.*

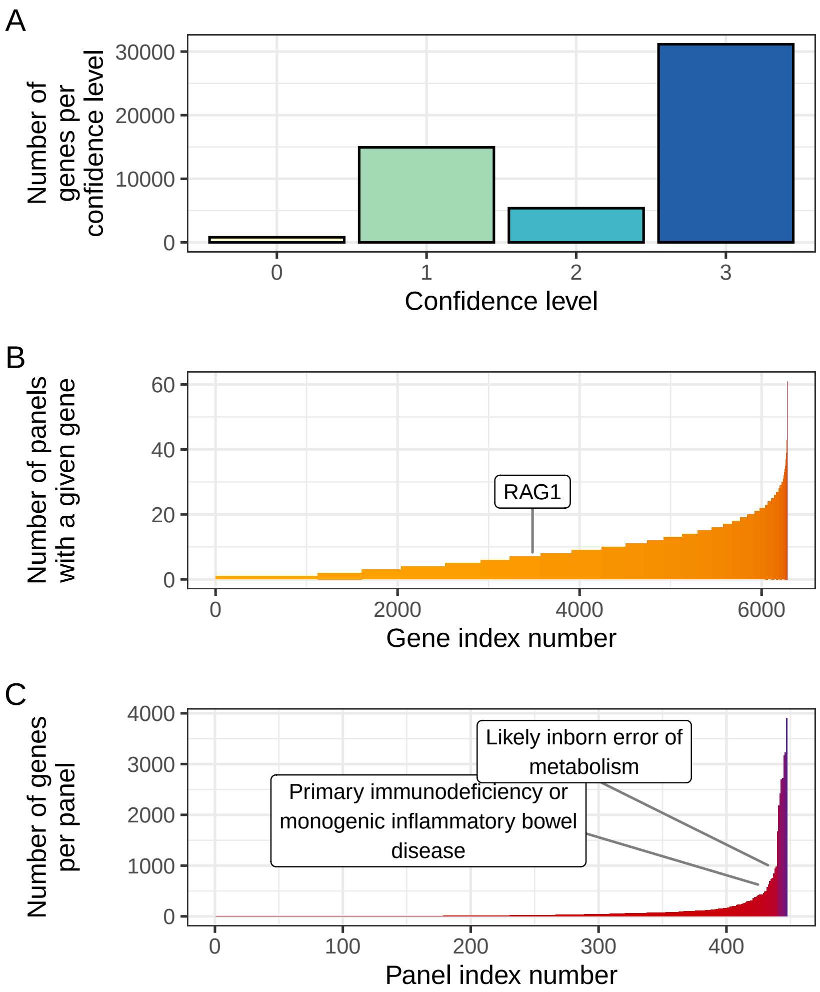

Figure S1: **Summary of gene confidence levels, reuse across panels, and panel sizes in the PanelAppRex dataset.** (A) Number of genes per confidence level as reported by **GE!**. (B) Number of panels in which each gene is included, with the example gene *RAG1* highlighted to demonstrate that it is present in 7 panels. (C) Number of genes per panel, with two representative panels annotated: PID/IEI and IEM.

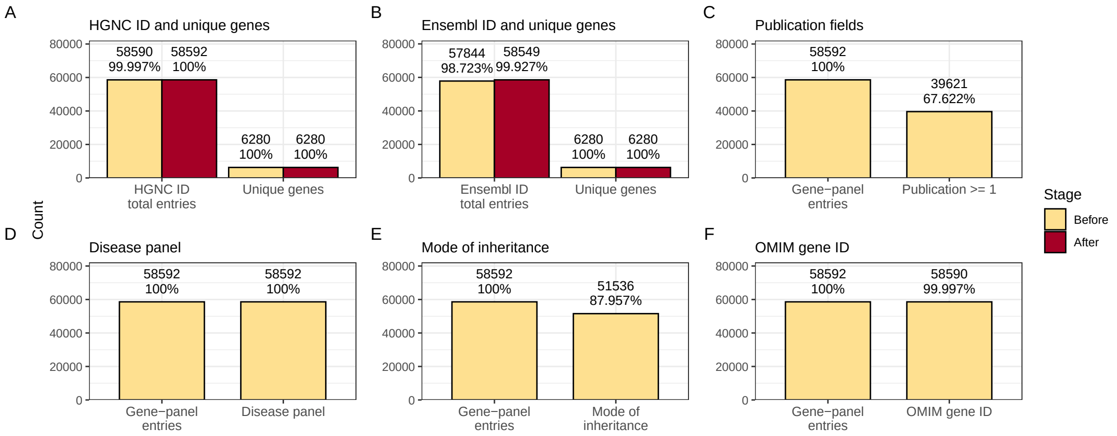

Figure S2: **Validation of completeness for major annotation fields in the core dataset.** (A) Entry-level completeness of HGNC identifiers before and after recovery using Ensembl biomaRt with HGNC symbol input, shown together with the total number of unique genes in the dataset as a reference. (B) Entry-level completeness of Ensembl gene identifiers before and after recovery using Ensembl biomaRt with HGNC symbol input, shown together with the total number of unique genes in the dataset as a reference. (C) Entry-level completeness of publication annotations, shown as the number of gene-panel entries compared to those with at least one linked publication. (D) Entry-level completeness of disease panel annotations, shown as the number of gene-panel entries with a gene and the number with a disease panel name. (E) Entry-level completeness of mode of inheritance annotations, shown as the number of gene-panel entries with a gene and the number with a recorded mode of inheritance. (F) Entry-level completeness of OMIM gene identifier annotations, shown as the number of gene-panel entries with a gene and the number with an OMIM gene identifier. For all panels the percentages are calculated relative to total gene-panel entries, except for panels A and B where the additional “unique genes” bars are calculated relative to the total number of unique genes. Entry-level refers to the initial merging of data from independent sources.

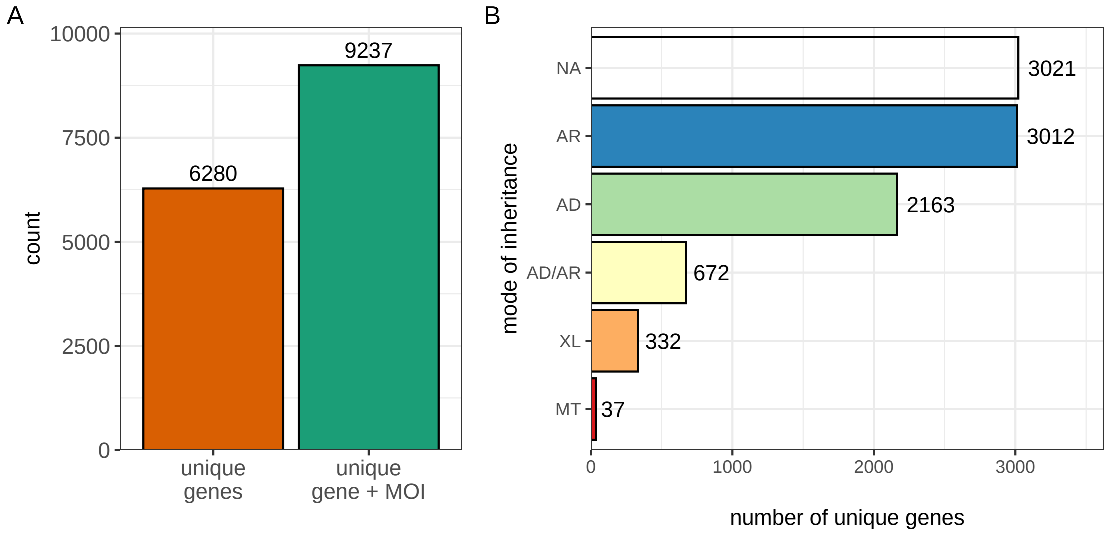

Figure S3: **Summary of mode of inheritance annotations in the PanelAppRex dataset.** (A) Counts of unique genes annotated with each MOI, based on non-redundant gene-MOI combinations. (B) Total number of unique genes and total number of gene-MOI combinations in the harmonised PanelAppRex dataset.

Performance

To assess whether PanelAppRex improves practical query performance beyond direct use of a comparable resource, we performed a cold-start benchmark against a best-case Genomics England (GE) PanelApp Application Programming Interface (API) workflow by re-using the first validation case query, "*SERPING1*, Factor XII, and edema" (**Figure** S4). We used the same query in both settings. This comparison is not fully like-for-like, because the GE PanelApp API does not accept the same phenotype-style query and return a ranked panel result directly. Instead, panel data must first be retrieved and assembled before ranking can begin. To make the comparison as favourable as possible to the API workflow, we deliberately included the known relevant panel from validation case study 1 and then increased the number of queried panels. In practice, a user would not usually know that panel in advance.

PanelAppRex local was faster at every tested subset size for time to final ranked result. The API workflow also required increasing numbers of panel-level requests in order to assemble the returned data before ranking could begin. Queries with more than 100 panel calls resulted in the GE API returning HTTP 429 rate-limiting fair usage errors, so larger panel sets could not be evaluated in this benchmark without introducing additional delays. In addition, the API comparator returned only panel content, whereas PanelAppRex provided the pre-integrated local dataset together with the annotations used elsewhere in the platform such as gene function and disease details.

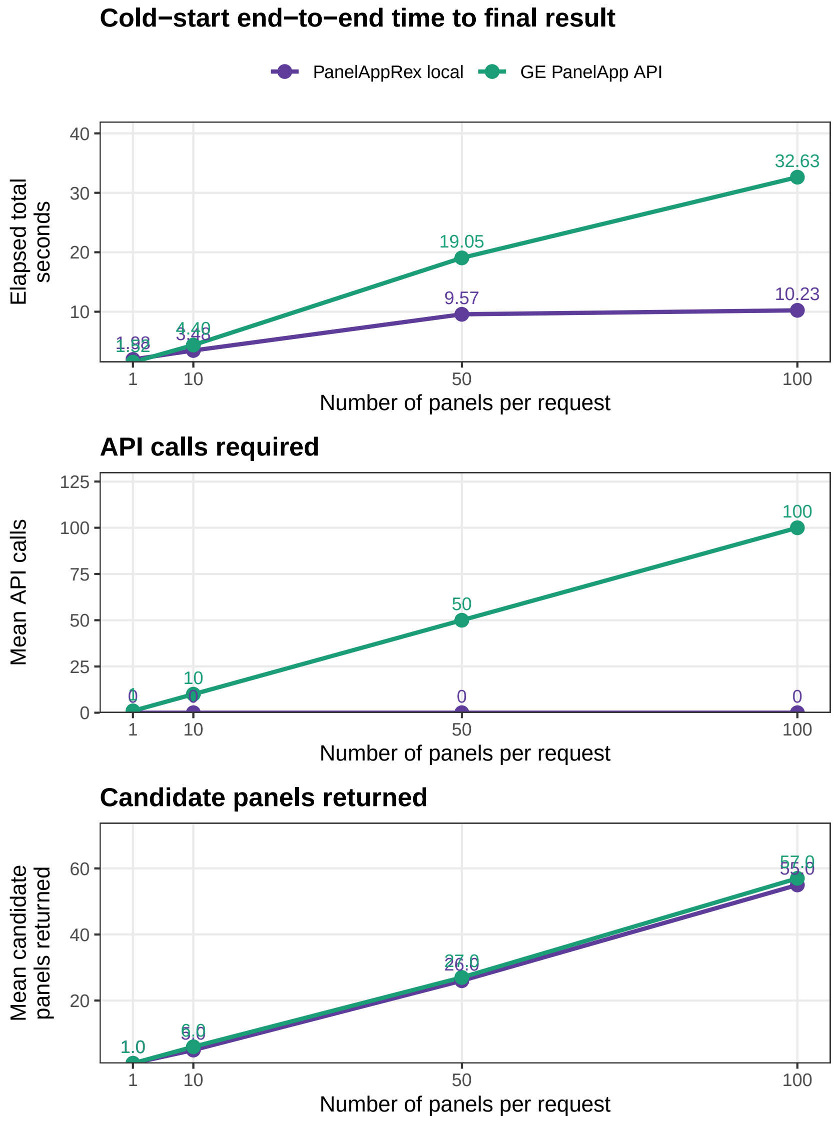

Figure S4: **Cold-start benchmark of PanelAppRex local against a best-case GE PanelApp API workflow.** The same clinical query from case study 1 was used in both settings. The x axis shows the number of panels in each benchmark set. For each run, the same growing panel set was used in both arms. The API comparator was deliberately made favourable by including the known relevant panel and then increasing the number of additional panels retrieved. (A) End-to-end elapsed time from query to final ranked result. (B) Mean number of API calls required to retrieve and assemble the queried GE panels before ranking. PanelAppRex local required no live API calls at query time. (C) Mean number of candidate panels returned after applying the input query to the retrieved panel set. For this speed benchmark, candidate panels were counted using at least one matching query term. After 150 panels in the benchmark set, the GE API returned HTTP 429 rate-limiting errors, so larger runs could not be completed in this benchmark.

Example AI-based Retrieval-Augmented Generation (RAG) summary

We used the core PanelAppRex dataset combined with RAG on the UniProtKB human proteome to generate AI-derived panel summaries that captured panel-specific mechanisms and phenotypes conditioned on disease context and gene composition. To demonstrate the results here, we show the first panel from the user interface, "Thoracic aortic aneurysm or dissection", which contains 65 genes. The AI-based RAG "Info" summary for this panel was:

Overview

This gene set primarily encompasses disorders of connective tissue and vascular smooth muscle leading to thoracic aortic aneurysm and dissection. Clinical features include progressive dilation and rupture risk of the thoracic aorta, arterial tortuosity, joint hypermobility, skin laxity, skeletal deformities such as scoliosis and pectus excavatum, ocular abnormalities like lens dislocation, and systemic manifestations of connective tissue fragility. Many syndromes represented show autosomal dominant inheritance with variable expressivity and overlap with Marfan, Loeys-Dietz, and Ehlers-Danlos syndromes.

Summary

- *Defects in collagen synthesis, processing, and fibril assembly compromise connective tissue strength, leading to vascular fragility and skeletal abnormalities.*
- *Impaired elastic fiber formation and maintenance disrupt aortic wall elasticity, predisposing to aneurysm and dissection.*
- *Dysregulated TGF-beta signaling pathways alter vascular smooth muscle cell function and extracellular matrix remodeling, contributing to arterial tortuosity and aneurysm formation.*
- *Smooth muscle contractile apparatus dysfunction affects vascular tone and structural integrity, increasing risk of thoracic aortic aneurysm.*
- *Phenotypes include thoracic aortic aneurysm and dissection, joint hypermobility, skin hyperextensibility, skeletal deformities (scoliosis, pectus excavatum), and ocular features such as ectopia lentis.*
- *Syndromic presentations overlap with connective tissue disorders including Loeys-Dietz syndrome, Ehlers-Danlos syndrome (vascular and classic types), and Marfan syndrome.*
